# The extracellular matrix and perineuronal nets in memory

**DOI:** 10.1038/s41380-022-01634-3

**Published:** 2022-06-27

**Authors:** James W. Fawcett, Marianne Fyhn, Pavla Jendelova, Jessica C. F. Kwok, Jiri Ruzicka, Barbara A. Sorg

**Affiliations:** 1 John van Geest Centre for Brain Repair, Department Clinical Neurosciences, University of Cambridge, Cambridge, CB2 0PY UK; 2 Centre for Reconstructive Neuroscience, Institute for Experimental Medicine CAS, Videnska 1083, Prague 4, Prague, Czech Republic; 3 Department of Biosciences, University of Oslo, Oslo, Norway; 4 School of Biomedical Sciences, University of Leeds, Leeds, LS2 9JT UK; 5 Robert S. Dow Neurobiology Laboratories, Legacy Research Institute, Portland, OR USA

**Keywords:** Neuroscience, Psychology

## Abstract

All components of the CNS are surrounded by a diffuse extracellular matrix (ECM) containing chondroitin sulphate proteoglycans (CSPGs), heparan sulphate proteoglycans (HSPGs), hyaluronan, various glycoproteins including tenascins and thrombospondin, and many other molecules that are secreted into the ECM and bind to ECM components. In addition, some neurons, particularly inhibitory GABAergic parvalbumin-positive (PV) interneurons, are surrounded by a more condensed cartilage-like ECM called perineuronal nets (PNNs). PNNs surround the soma and proximal dendrites as net-like structures that surround the synapses. Attention has focused on the role of PNNs in the control of plasticity, but it is now clear that PNNs also play an important part in the modulation of memory. In this review we summarize the role of the ECM, particularly the PNNs, in the control of various types of memory and their participation in memory pathology. PNNs are now being considered as a target for the treatment of impaired memory. There are many potential treatment targets in PNNs, mainly through modulation of the sulphation, binding, and production of the various CSPGs that they contain or through digestion of their sulphated glycosaminoglycans.

## Introduction

All components of the CNS are surrounded by a diffuse extracellular matrix (ECM) containing chondroitin sulphate proteoglycans (CSPGs), heparan sulphate proteoglycans (HSPGs), hyaluronan, various glycoproteins including tenascins and thrombospondin, and many other molecules that are secreted into the ECM and bind to ECM components. In addition, some neurons, particularly inhibitory GABAergic parvalbumin-positive (PV) interneurons, are surrounded by a more condensed cartilage-like ECM called perineuronal nets (PNNs). PNNs surround the soma and proximal dendrites as net-like structures that surround the synapses. Attention has focused on the role of PNNs in the control of plasticity, but it is now clear that PNNs also play an important part in the modulation of memory. In this review we summarize the role of the ECM, particularly the PNNs, in the control of various types of memory and their participation in memory pathology. PNNs are now being considered as a target for the treatment of impaired memory. There are many potential treatment targets in PNNs, mainly through modulation of the sulphation, binding, and production of the various CSPGs that they contain or through digestion of their sulphated glycosaminoglycans [1].

## Extracellular matrix biology

### Extracellular matrix

Extracellular matrix (ECM) refers to a collection of extracellular molecules that provides physical and biochemical support to cells. Studies on the ECM mainly focus on the intricate network of ECM formed by macromolecular assembly. The ECM in the central nervous system (CNS) is mainly composed of proteoglycans, glycosaminoglycans (GAGs) and glycoproteins such as tenascins and thrombospondin that interact with them [2]. Diffusion of molecules such as neurotransmitters, ions, guidance molecules, and metabolites are tightly regulated by this network.

Proteoglycans are a family of large ECM molecules whose basic structure comprises linear GAG chains covalently attached to a core protein. There are five types of GAGs, chondroitin sulphates (CS), heparan sulphates (HS), keratan sulphates, dermatan sulphates, and hyaluronan [3, 4]. Chondroitin sulphate proteoglycans (CSPGs) and heparan sulphate proteoglycans are the key proteoglycans in CNS function [3]. Research in the last three decades has elucidated the inhibitory functions of CSPGs in neurite extension, path-finding, plasticity and neural regeneration [5–9]. CSPG function is strongly influenced by the pattern of sulphation of the GAG chains, with 4-sulphated GAGs being inhibitory and 6-sulphated GAGs being permissive to axon growth and plasticity [10]. Synthesis of GAGs and their sulphation occurs in the Golgi, sulphation being determined by the activity of sulfotransferases that sulphate CS and HS chains in various positions on the constituent disaccharides [11]. In addition to being a key inhibitory molecule in the diffuse ECM, CSPGs around some classes of neurons also interact with other brain ECM molecules, self-assembling into aggregate structures called perineuronal nets (PNNs) [12–14]. The lectican family of CSPGs (also called aggrecan-family CSPGs) are found within PNNs, including aggrecan, brevican, neurocan, and versican [1].

While diffuse CNS ECM surrounds all structures in the CNS, perineuronal nets (PNNs) with a cartilage-like structure surround some classes of neurons. PNNs are reticular CSPG-containing ECM structures surrounding the soma and proximal dendrites of a subpopulation of CNS neurons and important for controlling neuroplasticity [1, 13]. Hyaluronan is synthesized by the transmembrane enzyme hyaluronan synthase (HAS), which anchors the nascent hyaluronan chains to the neuronal surface [15] together with ankyrin-R [16] and RPTPzeta/phosphacan [17]. The long hyaluronan chains provide a scaffold for the assembly of CSPGs, hyaluronan, hyaluronin and proteoglycan link proteins (Hapln), and tenascins [15, 18]. While binding of the N-terminal of CSPG to hyaluronan chains is stabilised by Hapln, the C-terminals of three CSPG molecules will link with the trimeric tenascin-R [19–21]. These interactions enable the formation of a stable PNN. However, the diffuse ECM also affects neuronal activity. Hyaluronan is a regulator of extracellular volume, and hyaluronan deficiency causes altered neuronal activity and seizures [22].

PNN components and their interactions with other ECM molecules such as OTX2, neuronal pentraxin 2 (Nptx2, also called Narp) and semaphorin3A (Sema3A), contribute to the functions of PNNs in neuroplasticity [23–27]. While OTX2 and Sema3A bind to 4,6 disulphated GAGs in the PNNs [23, 28], Nptx2 binds to both 4,6 sulphated GAGs and HA [29]. The soluble transcription factor OTX2 binds to PNNs and is internalised, leading to maturation of PV neurons and maintenance of PNNs in adulthood [23, 26]. Nptx2 is an activity-regulated protein that interacts with the extracellular domain of AMPA receptors to facilitate receptor clustering and insertion of GluR4 on the postsynaptic membrane of neurons, strengthening synaptic communication [24]. Removal of CS by chondroitinase ABC (ChABC) abolishes these effects of Nptx2. Sema3A is a chemorepulsive molecule, and prevention of its binding to PNNs reinstates ocular dominance and cerebellar plasticity in adult mice [30, 31]. Moreover, a recent study has also shown that Sema3A binding to CS GAGs induces rigidification of the CS matrix, which may alter the mechanical properties of PNNs and ultimately affect neuroplasticity [32]. Proteoglycans also exert effects through binding to a cell surface phosphatase, protein tyrosine phosphatase sigma (PTPσ), which can exert inhibitory effects from CSPGs and permissive effects from HSPGs [33].

### ECM and synapses

**Fig. 1:**
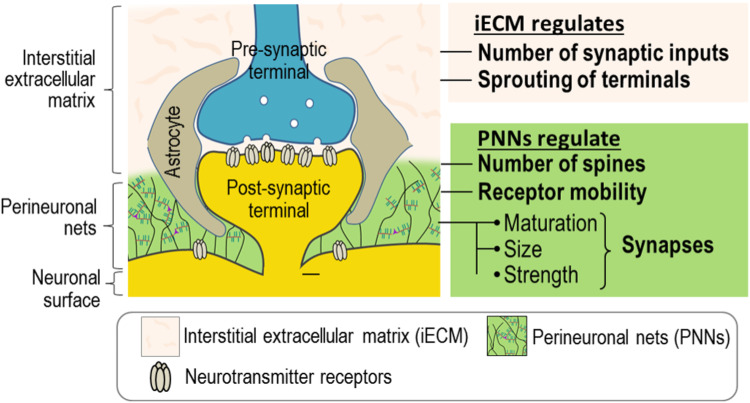
The CNS extracellular matrix. Synapses are tripartite structures involving pre-and postsynaptic structures and astrocytes. All synapses are embedded in interstitial extracellular matrix (iECM), which regulates the extracellular volume, but some synapses are also surrounded by a condensed form of ECM, the PNNs, consisting mainly of CSPGs attached to a hyaluronan backbone.

The substrate of memory is synaptic strength and connectivity. All synapses are embedded in ECM, either the general interstitial ECM found throughout the CNS or the specialized ECM of PNNs, where they can interact with CSPGs, HSPGs, tenascin-C, tenascin-R, thrombospondin, and laminins (Fig. 1). These molecules in turn bind and present other active molecules to neurons. Synaptic ECM molecules also interact directly with receptors and ion channels, modulating their migration and properties [34, 35]. The most-studied ECM molecules that affect synapses and memory are CSPGs. The ECM can be modified rapidly by the release of proteases [36], by microglial action and by internalization, all of which can be activated by memory events [37–39].

Experimentally, much of our knowledge of the effects of CSPGs and PNNs stems from their modification by digestion of the GAG chains using ChABC. At postsynaptic sites, ChABC enhances dendritic spine number and motility, while presynaptic terminals tend to show enhanced sprouting and synapse numbers [40–42]. In the perirhinal cortex and hippocampus, ChABC digestion increases inhibitory inputs to PV interneurons [43–45] while in the entorhinal cortex, ChABC reduces inhibitory inputs, and in V1 visual cortex, ChABC decreases both excitatory and inhibitory inputs to PV interneurons [46, 47]. The deep cerebellar nucleus, where PNNs surround most neurons, has been a fertile region for PNN research: Purkinje cell terminals sprout after ChABC digestion [48], and in the frontal cortex, the number of inhibitory connections to pyramidal cells is decreased [49]. Digestion of HA with hyaluronidase modulates synaptic function by increasing AMPA receptor mobility (reviewed in [50] and surface expression of NMDA receptors [35, 51]. PNN function can be modulated by removing individual components. Deletion of link proteins leads to fewer Purkinje synapses, decreases inhibitory transmission in the deep cerebellar nucleus [52], and facilitates long-term depression in the perirhinal cortex [53]. Manipulation of individual CSPG proteins can also affect synapses and synaptic function [34, 54]. A relevant function of CSPGs in PNNs is to present semaphorins to synapses; absence of semaphorin 4C (sema4C) prevents the increase in spine number during fear learning [55, 56]. Moreover, knockout mice deficient in PNN component tenascin-R have abnormal synapse formation and synaptic plasticity after injury [54, 57]. An important mechanism of plasticity is modification of the CNS ECM by activity-related release of metalloproteinases, which can cause rapid changes in PNNs in region of synapses, enabling local changes in synaptic properties [38, 58].

#### Electrophysiological effects

Digestion or transgenic attenuation of PNNs has various effects on electrophysiological properties that are dependent on brain region and type of PNN manipulation [59]. Most studies in the hippocampal CA1 region show that PNN degradation or attenuation decreases long-term potentiation (LTP)[60–65]. Similarly, LTP is also affected by CSPG sulphation, with loss of 6-sulphation causing loss of LTP in the perirhinal cortex and CA1 [45]. However, the effects of reducing PNNs may be dependent on the cell type surrounded by PNNs. For example, in the CA2, an area associated with social memory and which usually does not exhibit LTP, PNN depletion enables LTP [66]. Long-term depression (LTD) is also altered after PNN degradation, with both increases [67] and decreases [53, 60] reported. However, in general, there is an overall increase in network activity when PNNs are depleted or attenuated [53, 59, 68], possibly due to an overall reduction in inhibitory activity. In line with this, digestion of CSPGs in the primary visual cortex in rats or deletion of aggrecan in mice decreases inhibitory activity, causing the network to revert to an immature juvenile state and an increased level of activity-dependent plasticity [47, 69]. Enhanced learning of eyeblink conditioning is also observed after ChABC digestion in the deep cerebellar nucleus, although here it is induced by increased GABAergic transmission [31, 70].

The variable effects of PNN attenuation could be related to cell-specific expression patterns of PNNs. While PNNs predominantly enwrap PV inhibitory neurons in most brain areas, they surround excitatory neurons in the CA2 [66, 71]. Moreover, individual CSPGs have distinctive effects that may also contribute to the large variation in effects of PNN depletion. For example, brevican affects mainly excitatory synapses, regulating both AMPA receptors and potassium channels and the speed and duration of synaptic potentials, leading to impaired hippocampal LTP observed in brevican knockout animals [34, 65, 72]. In contrast, aggrecan affects inhibitory synapses on PV interneurons (Ruzicka unpublished observations), and mice deficient in neurocan showed impaired hippocampal LTP [64]. Tenascin-R deficient mice also have a disrupted PNN structure, impaired LTP in the hippocampus [63], and show reduced active zones in inhibitory synapses [73]. Lastly, animals deficient in tenascin-C show impaired L-type calcium channel-dependent LTP [74].

### Types of memory and memory models

#### Associative learning

To examine PNN function in associative memories, we focus on fear conditioning and eyeblink conditioning, two well-studied phenomena (see [75] for review). Fear and eyeblink memory are similar in that a conditioned stimulus (usually a tone, visual cue or context) is linked to an unconditioned stimulus; electric shock in the case of fear memory, a puff of air to the cornea for eyeblink memory. After a training period during which both stimuli are given simultaneously, the conditioned stimulus alone will cause animals to freeze (fear memory) or blink their eyes. The neural pathways differ, but both involve the auditory or visual pathways. Other forms of conditioning, such as that associated with drugs of abuse, are also discussed below.

#### Eyeblink conditioning

**Fig. 2:**
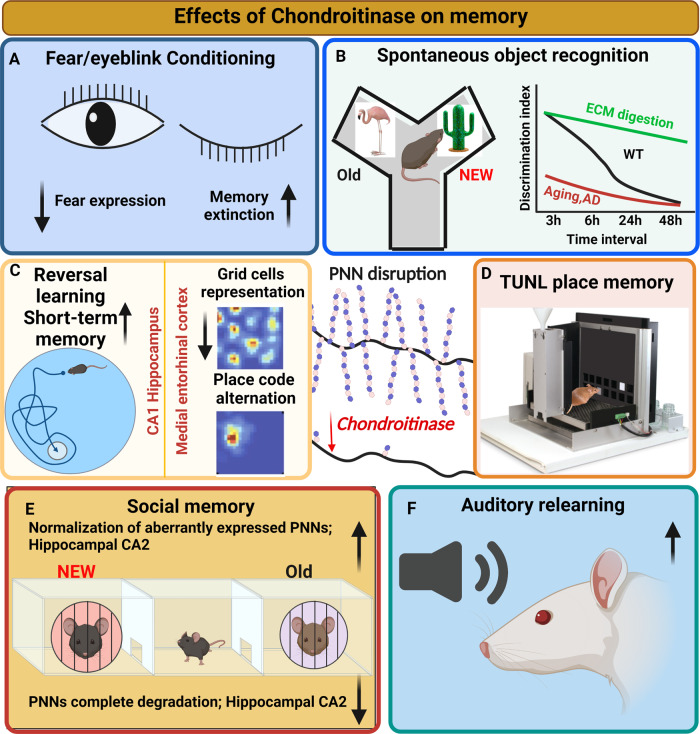
Memory effects of chondroitinase digestion. **A** Eyeblink conditioning learning is increased by ChABC to the cerebellar nuclei but persistence is decreased. In fear memory PNN digestion enables extinction. **B** Spontaneous object recognition is assessed in a Y-maze, animals distinguishing between familiar and non-familiar objects in the arms. After 5 min exposure to objects, memory gradually decays and by 24 h is mostly lost. ChABC treatment prolongs memory in young animals, restores it in models of Alzheimer’s and ageing. **C** The Morris water maze tests place learning: ChABC treatment increases reversal and short-term learning. Grid cells provide a map of the external world: the grid cell map is destabilized by ChABC treatment. **D** The trial-unique nonmatching-to-location assay (TUNL) is a hippocampus-dependent automated test of location memory. Memory acquisition is enhanced by ChABC treatment. **E** In normal animals, ChABC digestion impairs social memory, but in animals with defective social memory due to abnormal PNNs, digestion restores memory **F** ChABC digestion increases the agility of auditory relearning and decreases firing of fast-spiking neurons.

Delayed eyeblink conditioning is a type of associative conditioning that requires neurons in the deep cerebellar nuclei (DCN) [76], many of which are surrounded by PNNs [77]. The acquisition of eyeblink conditioning reduces the intensity of PNNs in the DCN, whereas longer training (to plateau levels) restabilizes PNN intensity [31]. Injection of ChABC [70] or viral vector-containing ChABC to provide long-term depletion of PNNs increases acquisition of eyeblink conditioning [31] but slightly decreases retention of this response when tested about three weeks later. This is consistent with reduced firing of these neurons, an increased number of inhibitory terminals and reduced excitatory terminals [31], and greater inhibition of DCN neurons [70]. The increased acquisition is in contrast to another study [78] that showed a reduced conditioned response and no change during extinction. The differences between studies may be due to differences in species, strength of the unconditioned stimulus, or the method of ChABC delivery. Sema3A is associated with PNNs around Purkinje cell terminals and may influence remodelling of synapses and in turn the impact of ChABC on eyeblink conditioning [56] (Fig. 2A).

#### Fear conditioning

Fear conditioning is often used as a model for posttraumatic stress disorder (PTSD), a psychiatric disorder characterized by hyperarousal, intrusive memories of traumatic events, and avoidance of reminders of those events [79]. While many studies focus on the basolateral amygdala (BLA), cortical regions also process threats associated with anxiety [80]. Studies in rodent models have focused on fear conditioning because PTSD in humans is believed to arise from abnormal activation of fear circuitry [81]. Fear memory was the first type of memory to be linked to PNNs and the ECM. Gogolla et al.[82] showed that PNN removal in the BLA in adult mice allowed for a subsequent extinction training to diminish expression of fear, similar to what occurs in juvenile mice prior to PNN development **(**Fig. 2A**)**.

Since then, other studies have shown that PNN degradation in the hippocampus, medial prefrontal cortex (mPFC), anterior cingulate cortex, BLA, or auditory cortex impairs the expression of fear conditioning [62, 83–85]. The effectiveness of ChABC implicates CSPGs, but digestion of hyaluronan also reduces fear memory retrieval [61]. Fear conditioning increases PNNs or mRNA encoding PNN components in the auditory cortex [84], hippocampus, and anterior cingulate cortex [62], and activates PNN-surrounded neurons [86]. PTPσ associates with PNNs and restricts plasticity by signalling through the receptor for brain-derived neurotrophic factor, TrKB [87, 88]. Sema4C, which also associates with PNNs (see above), is increased in the hippocampus and ACC following fear conditioning, and sema4C knockout mice show deficits in conditioned fear memory recall [55].

The formation and recall of fear memories and other associative memories involve many connected brain areas and need to be considered in the context of precisely timed brain oscillations synchronizing neural activity within and across brain regions. PV neurons are essential for these oscillations, and the impact of PNNs on learning and recall is likely to be tightly linked to their influence on the PV neuron network [89–92]. For instance, coherence (phase alignment) between theta oscillations in the secondary visual cortex (V2) and the BLA is necessary for successful recall of remote fear memories [93, 94]. Attenuation of PNNs in V2 weeks after training reduces theta coherency between BLA and V2 and prevents recall of a remote fear memory [94]. Moreover, Shi et al. [62] found that the increased theta power in the hippocampus and anterior cingulate cortex during fear conditioning is prevented by ChABC treatment, while overexpression of the PNN protein **hapln1** increases theta power.

### Spontaneous object recognition memory

The spontaneous novel object recognition (SOR) memory task measures discrimination between a novel and a familiar object presented at the same time. Novelty detection is an innate rodent behaviour that can be impaired during ageing or neurodegeneration [53, 95, 96]. The test is usually performed in a Y-maze, in which two test objects are placed in the Y arms. The times during which animals interact with the objects through whisking and smelling are measured; animals spend more time with objects that they perceive as novel. A variation is object-place memory testing in which objects are moved within a test arena, and animals recognize objects that have been moved to a new position. The brain regions that participate in the behaviour have been identified based on early gene *c-Fos* and *Arc* expression and lesion studies. For the Y-maze SOR test, a key brain area is the perirhinal cortex and the neighbouring visual association area TE: animals with lesions in these areas have impaired SOR [97]. Variations of the task where animals actively explore and dissociate the objects also involve the CA1 and CA3 areas of hippocampus whose rhythms are synchronised during generation of SOR memory [98, 99]. Object-place memory is primarily associated with the hippocampus, and may be preserved after perirhinal lesions [99]. Increased activity occurs in several regions during task performance, including the CA1 and CA3, perirhinal cortex, insular cortex, and medial PFC [98, 100]. Both hippocampus and perirhinal cortex are rich in PV interneurons enwrapped with PNNs, and PNNs are also present on some hippocampal pyramidal neurons [46, 101, 102]. Genetic or enzymatic attenuation of PNNs can increase synaptic transmission and facilitate long-term depression (LTD) in the perirhinal cortex [53] or CA1 region [67], and this correlates with enhanced recognition memory. Similarly, disaggregation of PNNs by genetic deletion of aggrecan shifts the population of PV inhibitory interneurons toward a juvenile-like plasticity state, accompanied by increased performance in the SOR memory task [69]. Another component of PNNs - brevican - regulates the localization of potassium channels and AMPA receptors on PV interneurons, and intact brevican is required for short-term, but not long-term SOR memories [34] (Fig. 2B).

### Spatial memory

Spatial memory is a form of episodic memory that depends on a distributed network of brain areas including the hippocampus, parahippocampal areas, and connected areas. The rich diversity of spatially modulated neurons in these areas - including place cells of the hippocampus [103] and grid cells [104, 105] of the medial entorhinal cortex as well as neurons estimating the distance to an object [106] or speed of the animal’s movement [107] - represent a rare window into neural correlates of complex behaviours and memories. Flexible spatial learning requires both dorsal and ventral hippocampus together with their connection to the mPFC [108], with the medial entorhinal cortex necessary for place navigation using a global reference frame [109].

The complexity of behaviours and the many brain regions involved makes it difficult to dissociate contributing elements. Nevertheless, regulation of PNNs and proper excitatory/inhibitory balance of these brain areas seem to be essential for spatial memory processing. Overexpression of ECM/PNNs in the CA1 area of hippocampus, either due to dysregulation of NPY-Y1 receptor signalling [110], targeted deletion of hyaluronan binding protein that mediates hyaluronan depolymerization (HYBID) [111], or defeat-induced persistent stress [112], leads to decreased spine density and deficits in spatial learning. In contrast, digestion of hippocampal PNNs with ChABC promotes re-learning of a once-trained Morris water maze task (Ruzicka et al., 2021, unpublished results). Similarly, significantly enhanced working memory and reversal learning in the Morris water maze task is found in TNR -/- global knockout mice [113] **(**Fig. 2C**)**. However, degradation of PNNs in medial entorhinal cortex, where PV positive neurons are enwrapped in particularly dense PNNs, destabilizes the grid cell networks leading to impaired representations of new environments [46]. The new representations also interfere with the map of familiar places. Following PNN removal in entorhinal cortex there are distorted spatial representations in downstream hippocampal neurons (Fig. 2C) [46]. This suggests that PNNs contribute to ensure a rigid grid cell network, which is essential for new representations to form, and that the heightened network plasticity caused by PNN removal interferes with stored spatial representations and perhaps memories.

The mPFC has an integrative role in object, place and time information [114, 115] as well as reward-regulated mechanisms of spatial learning [116, 117]. A robust approach to test the role of mPFC for spatial working memory is the mPFC-dependent trial-unique nonmatching-to-location assay (TUNL) task, a hippocampus-dependent automated test of location memory [118]. Infusion of ChABC into the mPFC improves performance on the touchscreen TUNL task [119] (Fig. 2D).

### Social memory

Social memory is explored using several experimental approaches. The basic principle is based on the propensity of rodents to investigate an unfamiliar subject more thoroughly than a familiar one. Commonly used is the habituation/dishabituation test, in which the animal repetitively explores the same subject animal with a time delay between explorations [120]. Another variant is the social discrimination paradigm [121], which has an initial exploration phase, but in the second phase, both familiar and novel subjects are presented at the same time. The task, usually performed in a three-chamber maze, shows high sensitivity for measuring social recognition in rodents [120, 122].

Social recognition memory is probably consolidated through the activation of cAMP response element-binding protein (CREB)-mediated gene expression in the hippocampus, mPFC, anterior cingulate cortex, and amygdala [123, 124]. Whereas the mPFC, anterior cingulate cortex, and amygdala are needed for coordination of brain activity during social interaction, the hippocampus serves as one of the mediators of social recognition memory ‘and as a connection hub between the various brain areas [123, 125]. The dorsal CA2 is the key centre for encoding, consolidation and recall phases of social memory [125–128]. CA2 also participates in social novelty discrimination [128] and modulates social aggression [129]. All the social memory associated regions are highly populated with PNN-surrounded PV neurons [46, 66, 130, 131]. Unusually in CA2 and the basolateral amygdala, PNNs are found around many excitatory pyramidal cells [47, 66], and calbindin-positive inhibitory interneurons [130]. PNNs play a distinct role in social memory, since mice with deficient social memory (BTBR mice) have atypical PNNs, and their degradation can partially restore social memory [132]. PNNs are usually associated with restriction of synaptic plasticity on inhibitory PV neurons, but in CA2, the PNNs also suppress LTP in excitatory synapses on pyramidal neurons [66]. However, PNNs in CA2 can also be permissive for inhibitory LTD (iLTD) in CA2, through maturation of PNNs and ErbB4 signalling at PV synapses [133]. This appears at the end of adolescence and correlates with social memory maturation. PNN degradation, in contrast, impairs social memory as well as iLTD induction [133, 134]. PNNs in CA2 are also upregulated during early postnatal exposure to an enriched environment, which opens the possibility of an early critical period synaptic plasticity in hippocampus [66] (Fig. 2E).

### Auditory plasticity/memory

The auditory pathway has tonotopic maps in the cortex and inferior colliculus that become refined during the critical periods for plasticity. As in other topographically arranged projections, PNNs contribute to the closure of these critical periods, with auditory experience and the diffusible transcription factor OTX2 which is a key factor in the initiation of PNN formation [135–137]. The timing of this transition at 3.5 years in deaf children is important for successful cochlear implants [138]. Learning of song in birds occurs either once or seasonally when PNNs are downregulated, and song is crystallized when PNNs appear [139]. In adult mammalian life, auditory learning is limited, but cortex-dependent auditory relearning regains the agility of the juvenile state after ECM digestion [140] (Fig. 2F). In the auditory cortex, the levels of brevican, which surrounds synapses in PNNs, changes over the course of auditory learning, with an initial decrease followed by a transient increase during consolidation [141]. Location of sounds is achieved in part by comparison of the timing of signals from each ear through the cochlear nucleus via the medial nucleus of the trapezoid body and lateral superior olive. In the trapezoid body there are massive synapses onto the principal cells called the Calyx of Held. These are specialized for very rapid and reliable transmission, and learning sound location requires these synapses. The CSPG brevican is enriched in the perisynaptic space of the Calyx, and knockout of brevican slows pre-to-postsynaptic action potential transmission and prolongs pre-and postsynaptic potentials [72].

The above-mentioned experiments describe the effects of attenuating PNNs, either naturally (as occurs during learning), by enzymatic degradation of PNNs, or by genetic disruption of PNN components, and suggest that PNNs may act as a brake on adult brain plasticity and perhaps learning and memory performance. It is important to note that abolishing PNNs by enzymatic approaches may not reflect processes occurring under physiological conditions in the brain. Rather, another suggestion is that learning induces slight changes to the ECM composition, either via incorporation of specific CSPGs [136, 142], metalloproteinase activity [143], or recycling of PNN components [144]. We are far from understanding the full complexity of this system. The outstanding richness and complexity of the ECM landscape, its components, and evolutionarily conserved endogenous regulators point to a fine-tuned regulation contributing to the brain’s ability to adapt and respond to a changing environment.

## Extracellular matrix and memory pathology

### Stress

**Fig. 3:**
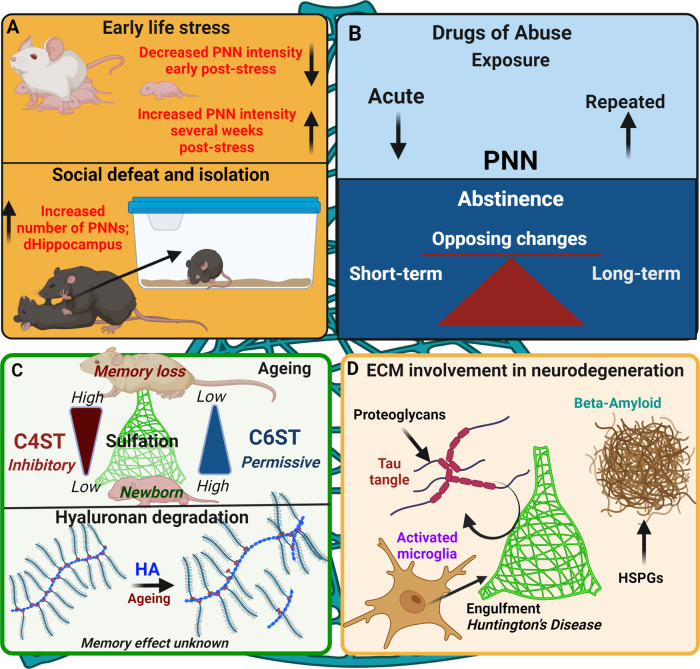
Effects of external events, neurodegeneration and ageing on the CNS extracellular matrix. **A** Stressful early life events, social isolation, social defeat and fear conditioning all have effects on numbers and intensity of PNNs. **B** Drugs of abuse have various and complex effects on PNN formation in different brain areas: please refer to the text. **C** During ageing, the sulphation pattern of PNNs changes, with a loss of permissive 6-sulphated CSPGs, leaving a predominance of inhibitory 4-sulphated forms. In addition, hyaluronan chains, which form the backbone of PNNs, become degraded into shorter fragments with unknown effects on memory. **D** The CNS ECM participates in neurodegenerative conditions. Proteoglycans participate in formation of tau tangles and beta-amyloidβ (Aβ) aggregates. In Huntington’s disease PNNs are engulfed by activated microglia.

Several studies have examined how acute and chronic stress exposures not involving fear conditioning influence PNNs. Spijker et al. [145] provide an excellent review on the impact of stress on PNNs. Although there are exceptions, in general, early life/adolescent stress reduces PNNs when examined early after stress, while these changes disappear or increases are found weeks after discontinuing stress. For example, decreases in PNNs around PV neurons are found in the hippocampus after chronic mild stress or maternal separation during adolescence, but an increase is observed several weeks post-stress [146–148]. In addition to time-dependent effects of stress, sex- and hemispheric-dependent differences have also been identified: early life chronic stress in rodents during postnatal days 1-10 increases BLA PNNs in males but not in females and show a hemispheric specificity [149]. In adults, often no changes or increases are found after discontinuing stress. For example, chronic stress increases PNN numbers in the mPFC and habenula [150]. Social defeat stress combined with social isolation for 2 months (producing a depression-like phenotype) increases the number of PNNs around PV neurons and PNN components in the dorsal hippocampus. Moreover, removal of PNNs with ChABC restores impaired memory and electrophysiological changes induced by this stress [112]. Consistent with the longer-term effects of stress on PNNs, another study on social defeat stress in young rodents showed biphasic effects, with decreases in PNN-enwrapped PV neurons and PNN components in the CA1 early after stress exposure but increases 2 months after stress exposure [151] (Fig. 3A). Overall, both early life and adult stress produce brain region-dependent changes in PNNs. The decreases in the intensity or number of PNNs found after early stress may reduce PV neuron activity or function, leading to enhanced output from brain regions such as the BLA that mediate fear responses [152].

### Drugs of abuse

Several studies have shown that drugs of abuse can either decrease or increase PNNs (see [153] for review). Several classes of drugs, including ethanol, nicotine, cocaine, and heroin, alter the intensity or number of PNNs in various brain regions, including the mPFC [154, 155], anterior cingulate cortex [131], orbitofrontal cortex [156], barrel cortex [157], insula [158], hypothalamus [159, 160], ventral tegmental area [156], and cerebellum [161–164]. Several examples of opposing direction of changes in PNNs are the following. (1) Acute vs. repeated cocaine injections produce opposite responses in PNN intensity in the mPFC [154]. (2) Extended exposure to cocaine self-administration increases PNN intensity in the cerebellum over abstinence time [162]. (3) Long-term abstinence times (2-3 weeks) or extinction from heroin self-administration reduces PNN components in the mPFC and/or nucleus accumbens, but even a short reinstatement session in these animals reverses PNN increases [165]. This latter finding suggests that the changes can be rapid (within several minutes). Other work examining the effects of cocaine and heroin self-administration also supports opposing effects of abstinence time on the number of PNNs in the mPFC (dorsal prelimbic and infralimbic, respectively) [166]. In the cerebellum, repeated cocaine exposures followed by an additional cocaine exposure one week later increase PNN intensity within DCN neurons [164], whereas similar treatment reduces PNN intensity 1 month later [161]. Cocaine conditioned place preference (CPP) training decreases PNN intensity in DCN neurons but increases PNN intensity in Golgi neurons, the latter of which is correlated with place preference. A binge model of alcohol in adolescents increases PNN intensity and PNN components in the orbitofrontal cortex in adult mice [167]. Extended ethanol drinking in adult mice increases PNN intensity in the mouse insular cortex after 6 weeks, but not after 1 week of exposure [158]. Short-term abstinence from nicotine self-administration also decreases PNN intensity in the orbitofrontal cortex and ventral tegmental area a few days after discontinuing exposure [156]. Thus, in general, short-term abstinence reduces PNNs, whereas long-term abstinence increases PNNs. However, as with stress, the changes are dependent on brain area, drug dose and class, exposure duration, and abstinence time from drug exposure **(**Fig. 3B**)**.

Several studies have demonstrated that removal of PNNs with ChABC alters behavioural responses to drugs. For example, removal of PNNs with ChABC in the mPFC or lateral hypothalamus prior to training for conditioned place preference (CPP) attenuates acquisition of the CPP memory [117, 159], and removal after CPP training also attenuates memory reconsolidation [117]. Removal of PNNs in the amygdala after training for morphine, cocaine CPP or heroin self-administration but before extinction reduces drug-primed reinstatement, but has no impact on reconsolidation, retrieval, or long-term morphine CPP memory [167]. Moreover PNN depletion in the lateral hypothalamus blocks cue-induced reinstatement in cocaine self-administering rats [160]. Depletion of the PNN component brevican in knockout mice enhances cocaine CPP 3 weeks after training, which is normalized by overexpressing this protein in the hippocampus prior to CPP training [168]. Extended ethanol exposure increases PNN intensity in the insular cortex, as mentioned, and removing PNNs in this brain region allows mice to become sensitive to the aversive effects of quinine added to ethanol, suggesting that increases in PNN intensity may contribute to the plasticity needed for compulsive ethanol seeking behaviour [169]. Interestingly, several of these studies found an effect only for drugs of abuse but not for non-drug rewards such as sucrose or food (e.g. [160, 166, 169],). Thus, the impact of PNN removal appears to be specific for plasticity induced by the learning/memory aspects of drugs of abuse.

Overall, stress or drugs of abuse bring about short-term changes in PNN numbers and/or intensity, while long-term increases in PNN may be related to loss of flexibility induced by subsequent natural stimuli, as previously considered for chronic exposure to stress [145] or drugs of abuse [170] **(**Fig. 3B**)**. The time of day PNNs are measured also may be critical due to daily rhythmicity in PNNs [171, 172]. PNN removal may enhance plasticity induced by weak stimuli or prevent metaplasticity induced by strong stimuli (stress or drugs of abuse). Whether these changes are beneficial or detrimental may depend on task demands, the neurons surrounded by PNNs (see [86]), the circuit that underlies task completion, and whether there is a need for sustained flexibility vs. stability after learning a particular task.

### Ageing

Cognitive impairment and memory loss are common changes in ageing. To maintain normal cognitive and memory functions in the face of scattered neuronal dysfunction, the nervous system needs a certain level of neuroplasticity to allow for adjustments in circuitry through changes in synaptic strength and the formation of new synapses [171].

Chondroitin sulphates and their sulphation pattern can determine whether or not there is memory loss in ageing. While chondroitin 4-sulphates (C4S) are inhibitory [5], chondroitin 6-sulphates (C6S) are more permissive to plasticity and regeneration [173, 174], and the balance between C6S and C4S regulates neuroplasticity. The sulphation pattern changes in the aged brain, having remained fairly constant since the end of the juvenile critical periods. Analysis of the PNN CSPGs in the aged rat and mouse brain showed that C6S almost disappears after 20 months while the level of C4S remains stable [45, 175] **(**Fig. 3C**)**. The effect of removing C6S on memory can be tested in transgenic mice with C6-sulfotransferase knockout, giving very low C6S levels. These animals showed a very early deficit in object recognition memory and spontaneous alternation memory as young as 3-months old, similar to the performance of 20-month-old aged mice [45]. The importance of C6 sulphation for memory was confirmed by virus-induced or transgenic expression of C6-sulfotransferase, leading to the restoration of the C6S level in aged mice and restoring or preventing age-related object recognition memory loss. As mentioned below in the neurodegenerative disease section, neutralisation of the inhibitory C4S with anti-C4S antibody restores object memory in a mouse tauopathy model [96]. These results indicate that the ratio of C6S:C4S is key to regulation of memory by PNNs.

Hyaluronan is another PNN component which demonstrates age-related changes in the brain. Long chain hyaluronan on the neuronal surface provides binding sites for the lectican family of CSPGs, which have a hyaluronan binding site, enabling hyaluronan to act as the backbone of the PNN [15]. Many studies show that the functions of hyaluronan depend on chain length. For example, low molecular weight hyaluronan is pro-inflammatory while high molecular weight hyaluronan is anti-inflammatory [176]. Changes in hyaluronan quantity have been reported in different pathological conditions such as ischemic and traumatic brain injury as well as in ageing [3, 177, 178]. A recent biochemical analysis of hyaluronan recovered from the PNNs in aged brains has shown its degradation into smaller fragments. This degradation has led to a release of other PNN components such as aggrecan into the soluble ECM [179]. Whether these age-related changes in hyaluronan affect memory is yet to be shown **(**Fig. 3C**)**.

### PNNs in genetic cognitive disorders

Rett syndrome is a neurodevelopmental disorder characterised by normal early development but then profound regression in cognitive, motor, and social function. It is caused by a loss-of-function mutation in the gene methyl-CpG–binding protein 2 (MECP2). The condition is associated with larger denser PNNs around PV interneurons in the cortex and many neurons in hippocampal CA2 (which mediates social behaviours), possibly due to decreased secretion of the metalloproteinase MMP-9. The increased PNN density causes loss of LTP in hippocampal neurons, which can be restored by ChABC digestion. In the cortex there is also an increase in the number and complexity of PNNs around PV interneurons in a Rett syndrome model, altering cortical excitability [102, 180]. Fragile X syndrome is a heritable condition causing intellectual disability and autism, modelled in mice by Fmr1 knockout. In these mice, there is a decrease in PNNs and impaired PV interneuron development in the cortex, hippocampus, amygdala and elsewhere. As well as general disability, the animals have a loss of tone-associated fear memory. The PNN decrease is associated with increased production of MMP-9, and genetic reduction or inhibition of MMP-9 production restores normal auditory responses and normalizes behaviour [181]. Schizophrenia, which is associated with various memory disorders, is also associated with a decrease in PNN numbers and density in the amygdala, thalamic reticular nucleus, entorhinal cortex and prefrontal cortex of patients [182]. Schizophrenia is linked to abnormalities in PV+ interneurons and an imbalance between glutamatergic and GABAergic transmission. A current hypothesis is that loss of the neuroprotective activity of PNNs renders the fast-firing PV+ oxidant-generating neurons vulnerable to oxidative stress [183].

### ECM memory in neurodegenerative disease

The main neurodegenerative disease associated with memory loss is Alzheimer’s disease, and most of the data linking the ECM to neurodegeneration apply to this condition. The ECM, in particular heparan sulphate proteoglycans (HSPGs) and CSPGs, are implicated in the progression of Alzheimer’s in several ways. In β-amyloid pathology, HSPGs bind to Aβ and are associated with plaques, affecting beta-amyloid precursor protein processing [184] and clearance [185]. Tau aggregation is promoted by proteoglycans [186], which are present in tangles, and are involved in the prion-like spread of tau pathology [187]. PNNs exclude tau pathology from the neurons that they surround, inhibiting tau uptake [188]. However, PNNs are themselves affected in Alzheimer’s disease [189] and in Huntington’s disease partly through engulfment by activated microglia [190, 191] **(**Fig. 3D**)**.

There is currently no treatment to prevent the progression of Alzheimer’s disease. However, the condition leads to the malfunction or death of scattered neurons, so functional compensation requires plasticity, some aspects of which, including spine and synapse loss, are impaired in Alzheimer’s, and some interventions aimed at synaptic transmission restore normal function [192]. From the perspective of the ECM, overall levels of plasticity can be restored to the levels normally associated with critical periods by manipulation of PNNs. Thus, digestion of PNNs in the perirhinal cortex of tauopathy mice restores object memory, ChABC digestion in Aβ pathology mice restores hippocampal function, and antibody blockade of the inhibitory 4-sulphated of PNN CSPGs restores object memory [96, 192, 193]. Modification of PNNs in Alzheimer’s disease could also come about through the action of activated microglia or secretion of metalloproteinases, both of which can occur in this condition [191, 194]. Reelin is an ECM-associated protein with effects on plasticity, and overexpression of this molecule restores memory in a tauopathy model [195]. Although much is yet to be understood about the role of PNNs in Alzheimer’s disease progression and cognitive decline, these investigations point important and mostly uncovered territory to understand this disease and identify much-needed new drug targets.

## Conclusion

The descriptions above show that the brain ECM, and particularly PNNs, play an important part in the regulation of memory and in memory pathology across a wide range of types of memory. This leads to the question of whether treatments that target PNNs could be useful for memory defects. At present, most of the evidence that memory can be modulated in useful ways comes from injections of ChABC into the CNS. This treatment is useful for proof-of-principle experiments, but is impracticable for long-term treatment of memory problems. However, there are many potential treatment targets in PNNs. An antibody that blocks inhibitory C4S has been effective at restoring memory in an Alzheimer’s model, and AAV-mediated expression of C6-sulfotransferase to reinstate C6S levels has restored memory in ageing. Other potential targets are small molecule inhibitors of C4S synthesis or activators of C6S synthesis, hyaluronan production by hyaluronan synthases, viral-mediated knockdown of aggrecan, and modulation or blocking of the diffusible transcription factor OTX2 [1, 196]. Future research holds promise for further insight into the function of the ECM in cognition and for the development of novel treatments.
